# Gene expression patterns associated with developmental transitions during somatic embryogenesis in pine

**DOI:** 10.1186/1753-6561-5-S7-P130

**Published:** 2011-09-13

**Authors:** Elena Carneros, Inmaculada Hernández, Silvia P Solana, Carmen Díaz-Sala, Dolores Abarca

**Affiliations:** 1 Department of Plant Biology, University of Alcalá, Alcalá de Henares, Madrid, 28871, Spain

## 

The low regeneration capacity of forest species is one of the major limitations for vegetative propagation [1]. The molecular mechanisms that determine the efficiency of clonal propagation programs via either adventitious organogenesis or somatic embryogenesis have not been established. For clonal propagation via somatic embryogenesis, the success of the process depends on an initial reprogramming step and on further developmental transitions involved in the maturation of somatic embryos [2] . The identification of candidate genes involved in the regulation of key steps of the regeneration processes is essential to generate tools and strategies to improve the success of clonal propagation programs in forest species.

The aim of this work is to identify new candidate genes potentially involved in the regulation of developmental transitions in somatic embryogenesis in pine. For that purpose, samples of embryogenic tissue from *Pinus radiata* D. Don at different stages of development were used: proliferative tissue (after 7 and 14 days from the last transference to fresh proliferation medium), somatic embryos at the beginning of differentiation and somatic embryos at cotyledonary stage [3,4].

Large-scale expression analysis using a microarray containing an EST collection enriched in auxin-induced genes, and several tissue-specific cDNA libraries from meristematic and embryonic tissues, were used for the identification of phase-specific candidate genes. Genes related to auxin signaling, regulation of gene expression, signal transduction, proliferation and embryo development were selected for further analysis. The expression of these candidate genes was confirmed by QRT-PCR. The information obtained from this work will open new ways of research on molecular mechanisms involved in developmental processes in conifers.

This work was funded by the Spanish Ministry of Science and Innovation (AGL-2008-05105-C02-01/FOR). Embryogenic lines were provided by C. Walter (Scion).
